# Infections of Leisure, 3rd ed.

**DOI:** 10.3201/eid1110.050828

**Published:** 2005-10

**Authors:** Richard Dawood

**Affiliations:** * Fleet Street Clinic, London, United Kingdom

**Keywords:** leisure, recreation, infection, book review

If you have ever thought about spending more time away from work, here is a book that could help change your mind. Infections of Leisure provides a detailed survey of the infective hazards associated with a wide range of human leisure activities and pursuits, from lazing on a beach to relaxing in a spa, dining out, or simply staying home and doing the gardening.

Now in its third edition, this book covers infections linked to salt and freshwater activities, camping and the outdoors, gardening, contact with animals, eating, foreign travel, sports, sexually transmitted diseases, body piercing, tattooing, and trekking to high altitude. The menu of topics is somewhat eclectic, and the balance between them is sometimes uneven, e.g., 30 pages on diseases associated with "Man's Worst Friend" (the rat), but only 20 pages on overseas travel. The result is nonetheless fascinatingly readable, even for the armchair practitioner.

On the subject of rats, I was intrigued to discover that 40,000 human rat bites are reported annually, and that *Rattenbisskrankheit*, or rat-bite fever in its various forms, has been noted clinically for >2,000 years. Bacterial zoonoses from domestic pets include salmonellosis from illegally kept turtles (i.e., those measuring <4 inches long). Both of these conditions have been the subject of recent case reports in the MMWR (*1**,**2*), confirming the continuing topicality of the book's contents.

There is much to whet the appetite of any connoisseur of bizarrely named syndromes, from "toxic sock" syndrome (pitted keratolysis caused by *Corynebacterium* in athletes) to "hot-foot" syndrome (plantar *Pseudomonas* folliculitis associated with abrasive swimming pool floors). But anyone looking for up-to-date information about more common conditions, from leptospirosis to Lyme disease, will find plenty of clear, concise, well-referenced material, contributed by experts in each field.

Leisure is a precious commodity, and this book remains a useful resource for anyone interested in knowing more about the pathogens that conspire against our pursuit of it, from the mundane to the truly outlandish.
